# Socio-Demographic Determinants and Behavioral Triggers of Tobacco Use: Insights From a Retrospective Study in Pune, India

**DOI:** 10.7759/cureus.111859

**Published:** 2026-07-01

**Authors:** Shams Ul Nisa, Shameeka Thopte, Vedika Pillai, Rashmi Sane, Abhijeet V Jadhav, Samir Khaire

**Affiliations:** 1 Oral Medicine and Radiology, Bharati Vidyapeeth Deemed to be University Dental College and Hospital, Pune, IND; 2 Oral and Maxillofacial Surgery, Government Dental College and Hospital, Mumbai, Mumbai, IND

**Keywords:** behavioural triggers, psychological, public health, smoking cessation, sociodemographic factors, stress, tobacco use, tobacco use disorder

## Abstract

Background

Tobacco use remains a major public health concern in India, with significant health, social, and economic consequences. Understanding patterns of use and behavioral triggers in clinical populations is essential for designing targeted cessation strategies.

Objective

To describe patterns of tobacco use, behavioural triggers, and nicotine dependence among patients attending a Tobacco Cessation Center (TCC) in Pune, India.

Methods

A retrospective descriptive analysis was conducted using anonymized intake records of 5,403 patients attending a TCC at a dental hospital between June 2017 and June 2023. Data on age, sex, marital status, occupation, type of tobacco use, behavioral triggers, nicotine dependence (assessed using the Fagerström test for nicotine dependence and its modified version for smokeless tobacco), quit attempts, and monthly expenditure were extracted. Descriptive statistics were used to summarize the data.

Results

The majority of participants were male (76.6%), married (85.2%), and belonged to the 41-60 years age group (42.1%). Smokeless tobacco was the most commonly used form of tobacco (71.9%), followed by smoking (27.0%), while dual use was reported by 1.1% of participants. Craving was the most frequently reported behavioral trigger for tobacco use (73.5%), followed by stress (17.7%) and peer influence (5.2%). Skilled professionals (39.2%) and unemployed individuals (32.3%) constitute the major occupational groups. Moderate nicotine dependence was the most commonly observed category (50.4%), followed by low dependence (33.8%) and high dependence (15.8%).

Conclusion

Tobacco use in this clinical population was predominantly observed among middle-aged males, with craving and stress reported as common triggers. The findings highlight the need for targeted, context-specific tobacco cessation strategies focusing on behavioral support and nicotine dependence management. However, given the descriptive design, no causal inferences can be made.

## Introduction

Tobacco consumption remains a major public health concern in India, contributing substantially to morbidity, mortality, and economic burden [1-3]. It is widely prevalent across socioeconomic groups, driven by the affordability, accessibility, and cultural acceptability of both smoking and smokeless forms of tobacco. Patterns of use differ across populations, with higher prevalence observed among specific demographic groups, including individuals influenced by social norms, peer behavior, and environmental exposure [4-6].

Tobacco is consumed in multiple forms, broadly categorized as combustible products (such as cigarettes and bidis) and non-combustible smokeless forms (including gutkha, khaini, and mishri), both of which are commonly used in the Indian context. These forms differ in their patterns of use, dependence potential, and associated health risks, particularly with respect to oral and systemic diseases. Despite ongoing public health measures, tobacco use continues to persist, highlighting the need for a better understanding of real-world usage patterns and behavioral triggers in clinical populations.

Efforts to reduce tobacco use have focused on multifaceted strategies, including community-based awareness campaigns, increased taxation, regulatory policies, and targeted interventions for high-risk groups [7-11]. However, much of the available evidence is derived from population-based surveys, with relatively limited data from Tobacco Cessation Centers (TCCs), where individuals actively seeking care may exhibit distinct behavioral patterns, dependence levels, and clinical presentations.

In this context, the present study aims to describe the patterns of tobacco use, behavioral triggers, and nicotine dependence among patients attending a Tobacco Cessation Center in Pune, India, thereby providing clinically relevant insights to support the development of targeted cessation strategies.

## Materials and methods

This retrospective descriptive study was conducted at the Tobacco Cessation Center (TCC) of a Dental College and Hospital in Pune, India, using data from 5,403 patient intake records collected between June 2017 and June 2023. All consecutive patients attending the TCC during the study period with a documented history of tobacco use were included. Patients without a history of tobacco use or with incomplete records for key variables were excluded from the analysis.

Data were extracted from standardized intake forms developed under the Dental Council of India TCC program. These forms captured demographic variables (age, sex, marital status, and occupation), referral source, tobacco use characteristics (type, form, frequency, behavioral triggers, and source of purchase), quit-related variables (previous cessation attempts and current quit status), and associated substance use (such as alcohol consumption).

Nicotine dependence was assessed separately using the Fagerström Test for Nicotine Dependence (FTND) for smokers and the Modified Fagerström Test for Smokeless Tobacco (FTND-ST) for smokeless tobacco users. Oral lesions were documented independently during routine clinical examination at the TCC using standard diagnostic criteria and recorded in the intake forms. Reported lesions included leukoplakia, oral submucous fibrosis, erythroplakia, tobacco pouch keratosis, and oral squamous cell carcinoma. Non-standard FTND values observed in the dataset were attributed to data entry variations in retrospective records and were categorized into standard dependence levels for analysis without modification of raw values.

Participants were categorized into four age groups: ≤20 years (Group A), 21-40 years (Group B), 41-60 years (Group C), and >60 years (Group D). Occupational categories were based on self-reported information recorded in the intake forms, and no standardized socioeconomic classification scale was applied.

Data were entered into a Microsoft Excel sheet (Redmond, USA) and analyzed using descriptive statistics. Continuous variables were summarized using mean and standard deviation, while categorical variables were presented as frequencies and percentages. No inferential statistical tests were performed, as the primary objective of the study was to describe patterns of tobacco use within the study population.

Ethical approval was obtained from the Institutional Ethics Committee of the Dental College and Hospital, Pune (Registration No.: EC/NEW/INST/2019/329). As this was a retrospective analysis of anonymized clinical records, informed consent was waived. All data were handled in accordance with ethical guidelines to ensure patient confidentiality.

## Results

The study analyzed data from 5,403 tobacco users attending a Tobacco Cessation Center. The distribution of socio-demographic and behavioral characteristics is presented below.

The majority of participants were married (85.2%, n = 4,604), followed by unmarried (12.3%, n = 663) and widowed individuals (2.5%, n = 136). Age-wise distribution showed that most participants were in the 41-60 years age group (42.1%, n = 2,275), followed by 21-40 years (37.2%, n = 2,010). Participants aged ≤20 years constituted 1.9% (n = 101), while those aged >60 years accounted for 18.8% (n = 1,017), as shown in Table 1.

**Table 1:** Age-wise distribution of tobacco use.

Age group (years)	n	%
≤20	101	1.9
21–40	2,010	37.2
41–60	2,275	42.1
>60	1,017	18.8
Total	5,403	100.0

Sex distribution indicated that males comprised 76.6% (n = 4,140) of the study population, while females accounted for 23.4% (n = 1,263), as represented in Table 2.

**Table 2:** Sex distribution of tobacco uses among participants.

Sex	n	%
Female	1,263	23.4
Male	4,140	76.6
Total	5,403	100.0

With respect to frequency of tobacco use, 30.7% (n = 1,660) reported use 0-5 times per day, followed by 6-10 times (18.8%, n = 1,016), 11-15 times (18.6%, n = 1,006), and 16-20 times (17.2%, n = 929). Only 14.7% (n = 794) reported using more than 20 times per day.

Duration of tobacco use indicated that 33.0% (n = 1,785) had been using tobacco for 6.1-10 years, while 31.3% (n = 1,692) reported use for 1.1-6 years. Only 1.4% (n = 73) reported a duration of less than one year. Occupational distribution showed that skilled professionals constituted the largest group (39.2%, n = 2,119), followed by unemployed individuals (32.3%, n = 1,747), physical laborers (24.2%, n = 1,307), and students (4.3%, n = 230), as shown in Table 3.

**Table 3:** Distribution of tobacco use according to occupation. Occupational categories are based on self-reported data; no standardized socioeconomic classification scale was applied.

Occupation	n	%
Student	230	4.3
Unemployed	1,747	32.3
Skilled professional	2,119	39.2
Physical labourer	1,307	24.2
Total	5,403	100.0

**Figure 1:**
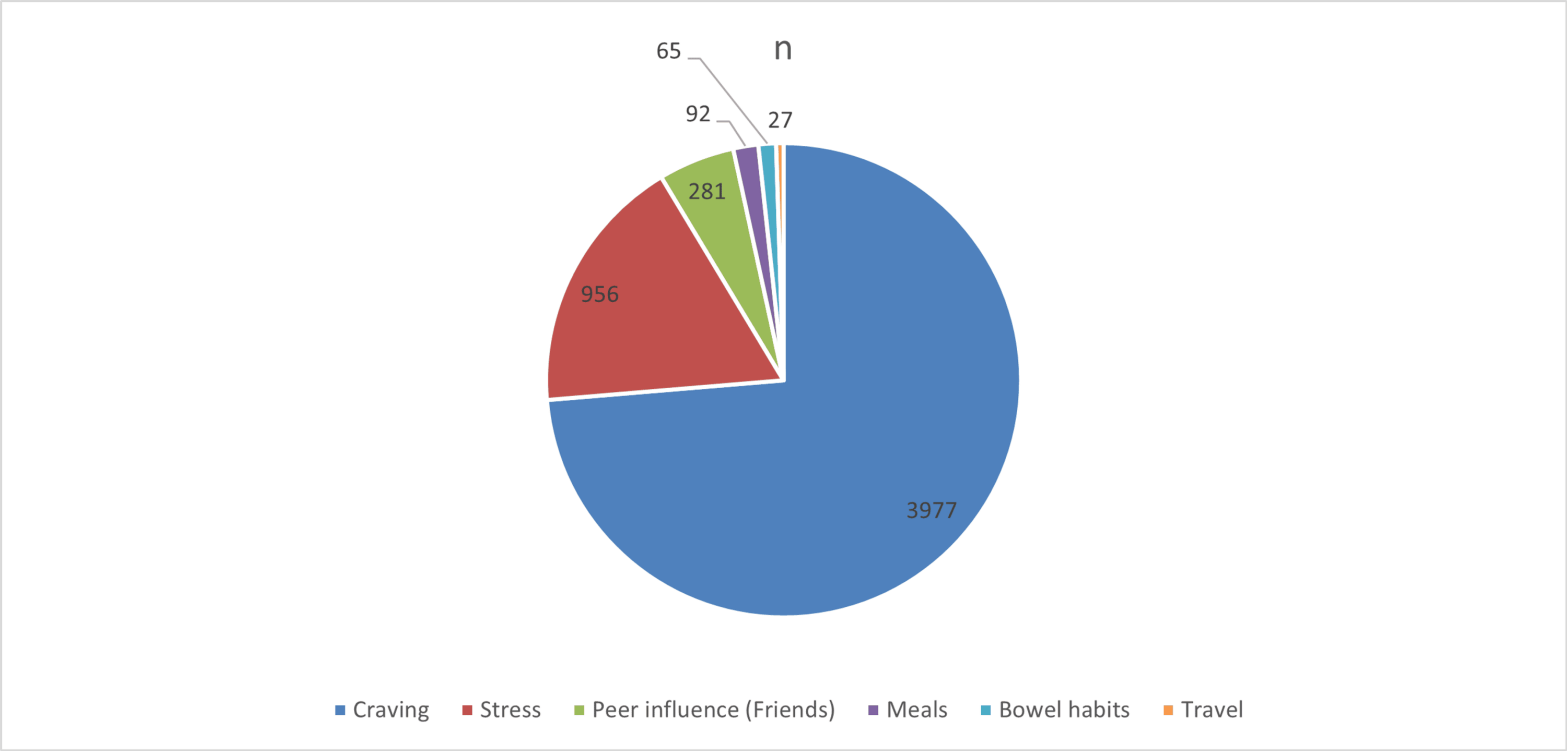
Distribution of participants according to self-reported reasons for tobacco use.

Behavioral triggers for tobacco use showed that craving was the most commonly reported factor (73.5%, n = 3,977), followed by stress (17.7%, n = 956) and peer influence (5.2%, n = 281). Other reported factors included meals (1.7%, n = 92), bowel-related habits (1.2%, n = 65), and travel (0.5%, n = 27), shown in Figure 1.

**Figure 2:**
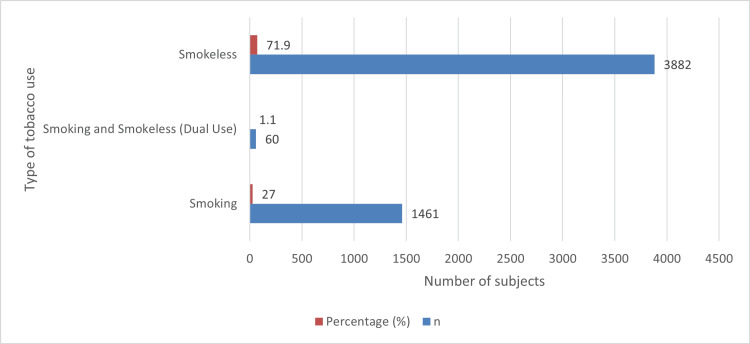
Distribution of participants according to type of tobacco use.

Regarding the type of tobacco use, smokeless tobacco was the most prevalent form (71.9%, n = 3,882), followed by smoking (27.0%, n = 1,461). Dual use of both smoking and smokeless tobacco was reported by 1.1% (n = 60) of participants, as depicted in Figure 2.

Monthly expenditure on tobacco indicated that 90.2% (n = 4,873) of participants reported spending ₹0-1,000, while 8.2% (n = 443) spent ₹1,001-2,000. A smaller proportion reported expenditure of ₹2,001-4,000 (0.9%, n = 50) and ≥₹4,000 (0.7%, n = 38), as shown in Table 4.

**Table 4:** Monthly expenses on tobacco. Income data were not available; affordability could not be assessed.

Monthly expense (₹)	n	%
0–1,000	4,873	90.2
1,001–2,000	443	8.2
2,001–4,000	50	0.9
≥4,000	38	0.7
Total	5,403	100.0

Alcohol consumption was reported by 10.0% (n = 542) of participants, while 90.0% (n = 4,861) reported no alcohol use.

Nicotine dependence assessment using the Fagerström Test demonstrated that moderate dependence was the most commonly observed category (50.4%, n = 2,724), followed by low dependence (33.8%, n = 1,824) and high dependence (15.8%, n = 855), as depicted in Table 5.

**Table 5:** Distribution of nicotine dependence levels based on reported FTND scores. Some non-standard FTND values (e.g., decimal scores and values exceeding the conventional range of 0–10) were observed in the dataset. For analysis, scores were grouped into standard dependence categories based on approximate ranges.

Nicotine dependence level	Score range*	n	%
Low dependence	0–3	1,824	33.8
Moderate dependence	4–6	2,724	50.4
High dependence	≥7	855	15.8
Total		5,403	100.0

**Figure 3:**
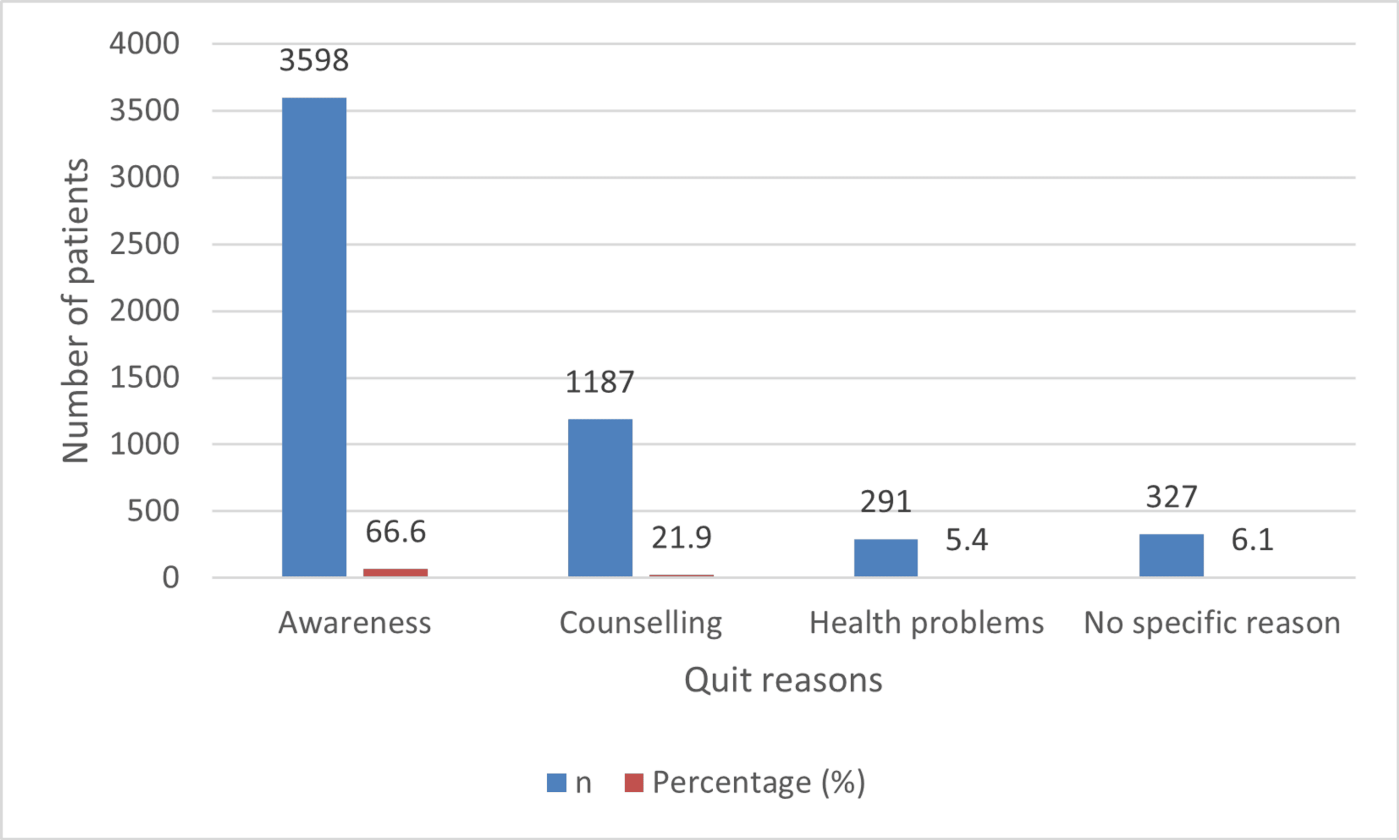
Distribution of participants according to reported reasons for quitting tobacco.

Reasons for quitting tobacco included awareness (n = 3,598), counselling (n = 1,187), health problems (n = 291), and no specific reason (n = 327), as shown in Figure 3.

**Figure 4:**
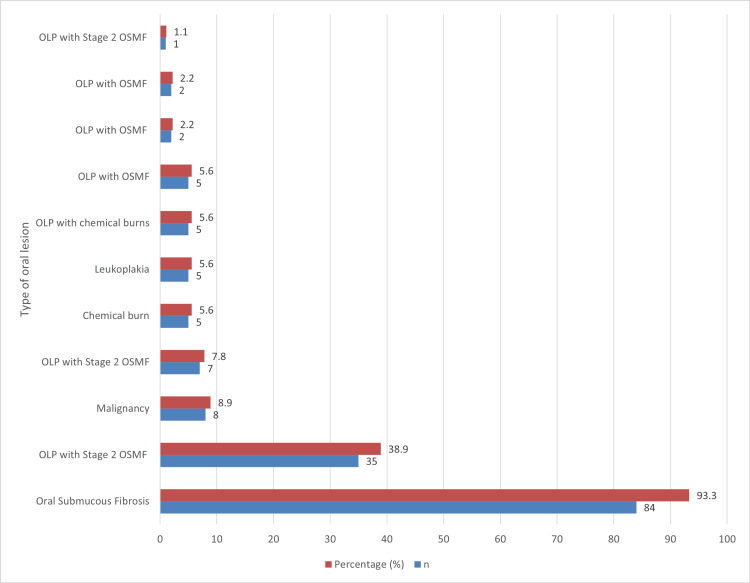
Frequency distribution of oral lesions among tobacco users.

Oral lesion assessment revealed that oral submucous fibrosis was the most commonly recorded lesion (n = 84), followed by OLP with Stage 2 OSMF (n = 35), malignancy (n = 8), OLP with Stage 2 OSMF variants (n = 7), leukoplakia (n = 5), chemical burns (n = 5), OLP with OSMF (n = 5), OLP with OSMF variants (n = 2 each), and OLP with Stage 2 OSMF variant lesions (n = 1), as shown in Figure 4.

The distribution of dependents showed that participants with 3-4 dependents constituted the largest group (37.2%, n = 2,012), followed by participants with 1-2 dependents (27.3%, n = 1,471). Participants with ≥5 dependents accounted for 19.7% (n = 1,066), while those with no dependents constituted 15.8% (n = 854), as shown in Table 6.

**Table 6:** Distribution of number of dependents among participants.

Number of dependents	n	%
0	854	15.8
1–2	1,471	27.3
3–4	2,012	37.2
≥5	1,066	19.7
Total	5,403	100.0

## Discussion

The present study describes patterns of tobacco use across socio-demographic and behavioral variables among a large clinical sample of 5,403 participants attending a Tobacco Cessation Center. The findings provide insight into the distribution of tobacco use and reported behavioral triggers within a help-seeking population.

A high proportion of participants in the present study were married (85.2%). While this finding is consistent with previous reports describing tobacco use in adult populations, the present study did not assess psychosocial variables such as marital stress or relationship dynamics; therefore, no causal interpretation can be made regarding the role of marital status in tobacco use [12]. The predominance of participants in the 41-60 years age group aligns with existing literature indicating higher tobacco use in middle-aged adults, although the present findings are limited to descriptive distribution within the study population [13].

The majority of participants were male (76.6%), which is consistent with previously reported gender-based differences in tobacco use in India. Prior studies have attributed such patterns to sociocultural norms and differential exposure; however, these factors were not directly assessed in the present study [14,15].

Occupational distribution showed a higher representation of skilled professionals and unemployed individuals. While earlier literature has linked occupational stress and socioeconomic conditions with substance use behaviors, the present study did not evaluate these associations analytically, and the findings should be interpreted as distributional patterns rather than evidence of causation [16].

Craving (73.5%) and stress (17.7%) were the most commonly reported behavioral triggers for tobacco use. These findings are consistent with existing literature suggesting the role of psychological and dependence-related factors in tobacco consumption. However, as these variables were self-reported and not measured using validated psychological scales, the interpretation should be limited to reported patterns rather than confirmed behavioral mechanisms [17].

Despite the predominance of moderate nicotine dependence levels, only a small proportion of participants (9.1%) reported previous quit attempts. This finding is in line with earlier studies that have highlighted gaps in cessation behavior and utilization of support services. Structured cessation programs combining counselling and pharmacological support have been shown to improve quit outcomes [18].

Most participants reported monthly expenditure below ₹1,000. However, in the absence of income-related data, the affordability of tobacco products could not be determined. Previous studies have highlighted the role of price in influencing tobacco consumption, but such relationships were not evaluated in the present study [19,20].

The presence of oral lesions such as leukoplakia and oral submucous fibrosis in the study population reflects the clinical burden associated with tobacco use. These findings emphasize the importance of routine oral screening in tobacco cessation settings.

Overall, the study provides a descriptive overview of tobacco use patterns within a clinical population. The findings may assist in identifying priority areas for intervention, particularly in relation to behavioral triggers and cessation support.

Limitations

This study has several limitations. First, it is a retrospective analysis based on clinical intake records, which may be subject to recall bias and data inconsistencies, particularly for self-reported variables such as triggers and expenditure. Second, the study population consists of individuals attending a Tobacco Cessation Center, representing a help-seeking group; therefore, the findings may not be generalizable to the broader community of tobacco users. Third, the absence of inferential statistical analysis limits the ability to assess associations or predictors of tobacco use. Additionally, important variables such as income level, detailed psychological assessments, and longitudinal follow-up data were not available, restricting deeper interpretation of behavioral patterns.

Future prospects

Future research should focus on prospective and community-based study designs incorporating detailed socioeconomic and psychological variables to better understand tobacco use behavior. The inclusion of validated tools for assessing stress, dependence, and behavioral triggers would improve data accuracy. Analytical studies employing inferential statistics are needed to evaluate associations and predictors of tobacco use. Additionally, longitudinal studies examining cessation outcomes, relapse rates, and the effectiveness of targeted interventions would provide valuable evidence for policy and clinical practice.

## Conclusions

This study highlights the multifaceted and complex nature of tobacco use, emphasizing the interplay of socio-demographic, behavioral, and economic determinants. The findings underscore the need for targeted and multifaceted interventions aimed at populations most affected, particularly middle-aged, married, and male individuals. Craving was the most frequently reported trigger, followed by stress. Implementing easily accessible, affordable cessation programs that offer pharmacological aids, individual counselling, and group support sessions can significantly enhance quit rates. These programs should be tailored to address gender-specific and occupation-related challenges to ensure inclusivity and effectiveness. By addressing the socio-demographic and behavioral determinants highlighted in this study, policymakers and public health professionals can implement strategies that reduce the prevalence of tobacco use and its associated health risks, contributing to healthier societies and economies.
